# Genetic Diversity in *Salmonella enterica* in Outbreaks of Foodborne and Zoonotic Origin in the USA in 2006–2017

**DOI:** 10.3390/microorganisms12081563

**Published:** 2024-07-31

**Authors:** Eija Trees, Heather A. Carleton, Jason P. Folster, Laura Gieraltowski, Kelley Hise, Molly Leeper, Thai-An Nguyen, Angela Poates, Ashley Sabol, Kaitlin A. Tagg, Beth Tolar, Michael Vasser, Hattie E. Webb, Matthew Wise, Rebecca L. Lindsey

**Affiliations:** 1 Association of Public Health Laboratories, Bethesda, MD 20814, USA; 2 Centers for Disease Control and Prevention, Atlanta, GA 30333, USA

**Keywords:** *Salmonella*, outbreak, genetic, diversity

## Abstract

Whole genome sequencing is replacing traditional laboratory surveillance methods as the primary tool to track and characterize clusters and outbreaks of the foodborne and zoonotic pathogen *Salmonella enterica* (*S. enterica*). In this study, 438 *S. enterica* isolates representing 35 serovars and 13 broad vehicle categories from one hundred epidemiologically confirmed outbreaks were evaluated for genetic variation to develop epidemiologically relevant interpretation guidelines for *Salmonella* disease cluster detection. The Illumina sequences were analyzed by core genome multi-locus sequence typing (cgMLST) and screened for antimicrobial resistance (AR) determinants and plasmids. Ninety-three of the one hundred outbreaks exhibited a close allele range (less than 10 allele differences with a subset closer than 5). The remaining seven outbreaks showed increased variation, of which three were considered polyclonal. A total of 16 and 28 outbreaks, respectively, showed variations in the AR and plasmid profiles. The serovars Newport and I 4,[5],12:i:-, as well as the zoonotic and poultry product vehicles, were overrepresented among the outbreaks, showing increased variation. A close allele range in cgMLST profiles can be considered a reliable proxy for epidemiological relatedness for the vast majority of *S. enterica* outbreak investigations. Variations associated with mobile elements happen relatively frequently during outbreaks and could be reflective of changing selective pressures.

## 1. Introduction

Non-typhoidal *Salmonella* subspecies causes an estimated 1 million cases of illness annually in the United States of America (USA), accounting for approximately 11% of all reported foodborne illnesses in the country, second only to the Norovirus [1]. Approximately 27% of salmonellosis cases are hospitalized and 0.5% result in death. The species *Salmonella enterica* (*S. enterica*) shows a tremendous amount of diversity just in terms of the over 2500 serovars described. However, the top 20 most common serovars account for 70% of all human isolates reported in the USA [2]. There are recognized differences in the genetic variation within the different *Salmonella* serovars; some serovars are known to be extremely clonal such as the serovar Enteritidis. For instance, epidemiologically unrelated strains of serovar Enteritidis often differ by only a couple dozen alleles or single nucleotide polymorphisms (SNPs) [3]. In contrast, other serotypes are polyphyletic, such as the serovars Muenchen, Newport, Saintpaul, Senftenberg, and Thompson, where strains of the same serovar can differ from each other by thousands of alleles or SNPs and reside in different lineages of the phylogenetic tree [4]. 

Active laboratory-based surveillance of *Salmonella* and other major bacterial foodborne pathogens in the USA is conducted by PulseNet, the decentralized network of over 80 state, county, city, agricultural, environmental, and federal laboratories. When PulseNet was established in 1996, the gold standard method used for molecular surveillance and cluster detection was pulsed-field gel electrophoresis (PFGE) subtyping. With the increased availability of affordable benchtop sequencing instrumentation and reagents, the complete replacement of PFGE with whole genome sequencing (WGS) as the gold standard method in PulseNet was completed for all PulseNet pathogens in 2019 [5]. 

During the era of PFGE-based surveillance, clusters of illness were detected based on indistinguishable PFGE patterns that rose in incidence above the expected five-year average baseline within the last 60 (*Salmonella*, *Escherichia coli*, *Campylobacter*, *Vibrio*) or 120 (*Listeria*) days [5]. Additional PFGE patterns were added to the outbreak case definitions if the epidemiological information and/or additional subtyping data warranted inclusion [6,7]. For real-time surveillance, PFGE information was expected to be uploaded to the national databases within four working days upon receipt of the isolate at the PulseNet laboratory [5]. However, additional subtyping information, such as the complete antigenic formulas for the serovars, antimicrobial susceptibility testing, and multiple-locus variable-number tandem repeat analysis (MLVA) were usually available at a much later date and only for a subset of isolates. 

WGS-based testing has revolutionized laboratory surveillance not only with the availability of higher resolution data, but also with the timely access to more complete data, such as predicted antimicrobial resistance (AR) profiles, on all or nearly all isolates included in outbreak investigations. However, large amounts of high-resolution data can pose interpretation problems, particularly in terms of defining disease clusters that are meaningful for epidemiological investigations. Our past experiences with legacy subtyping methods, such as PFGE and MLVA, have demonstrated that the amount of genetic variation within an outbreak can vary based on the length and the type of the outbreak (e.g., food versus zoonotic source, clonal versus polyclonal contamination). Other potential factors influencing genetic variation include the genetic stability of the etiologic organism itself, the amount of person-to-person transmission present within the outbreak, and the sensitivity, reproducibility, and stability of the typing method used to evaluate the relatedness [8].

The aim of this study was to retrospectively evaluate the genetic diversity within well-characterized *S. enterica* outbreaks using WGS and the PulseNet USA and National Antimicrobial Resistance Monitoring System (NARMS) data analysis workflows to establish the optimized cluster detection guidelines.

## 2. Materials and Methods

### 2.1. Selection of Outbreaks and Bacterial Strains

One hundred foodborne and zoonotic (live animal contact) outbreaks caused by non-typhoidal *S. enterica* subsp. *enterica* that occurred in the USA between 2006 and 2017 were included in the study. The outbreaks were detected by PulseNet based on indistinguishable PFGE patterns [9] and confirmed by epidemiological investigations. Only outbreaks with confirmed or suspected vehicles were included in the analysis. It is worth noting that the outbreaks included in this study may not be representative of all outbreaks that took place in the USA during the study period due to possible bias in the isolate selection process. The isolate selection criteria were primarily based on the availability of the isolates and accessibility to reliable epidemiological data for the sequenced isolates. For visualization purposes, the outbreak food vehicles were placed in 12 broad categories based on the Interagency Food Safety Analytics Collaboration (IFSAC) reporting levels 1, 2, and 3 [10]. All outbreaks associated with live animal contact were placed in a 13th category called “zoonotic”. Both the broad reporting categories and the exact vehicle types are listed in Table 1. Polyclonal outbreaks caused by multiple serovars were excluded from the study, as well as outbreaks in which person-to-person transmission was likely the major mode of transmission. Between 2 and 38 isolates with confirmed epidemiological information were sequenced from each outbreak to examine the genetic diversity within the outbreak. This resulted in a total of 438 sequences representing 35 different serovars. The isolates were obtained from the Centers for Disease Control and Prevention (CDC, Atlanta, GA, USA) and the state, county, and city health department culture collections, and fall under the CDC Institutional Review Board approved protocol no. IRB7172.

### 2.2. Whole Genome Sequencing (WGS)

Whole genome sequencing was performed either by CDC or by the PulseNet participating laboratories in their respective facilities. The strains were grown overnight on tryptic soy agar plates with 5% sheep blood (ThermoFisher Scientific, Waltham, MA, USA). DNA was extracted using the DNeasy Blood and Tissue kit (Qiagen, Germantown, MD, USA) and the quality was assessed as described in the PulseNet standard operating procedure [11]. DNA libraries were prepared by using either the Illumina Nextera XT (Illumina Inc., San Diego, CA, USA) or the Illumina DNA Prep (Illumina Inc.) library preparation kits according to the PulseNet procedures [12,13], followed by sequencing on the MiSeq (Illumina Inc.) [14]. Alternatively, DNA libraries were prepared using the NEB Next Ultra II kit (New England Biolabs, Ipswich, MA, USA) according to the manufacturer’s instructions, followed by sequencing on the HiSeq (Illumina Inc.). Raw sequences were made available on the Sequence Read Archive of the National Center for Biotechnology Information. The accession numbers are listed in the Supplemental Table S1. 

### 2.3. Data Analysis

**Figure 1:**
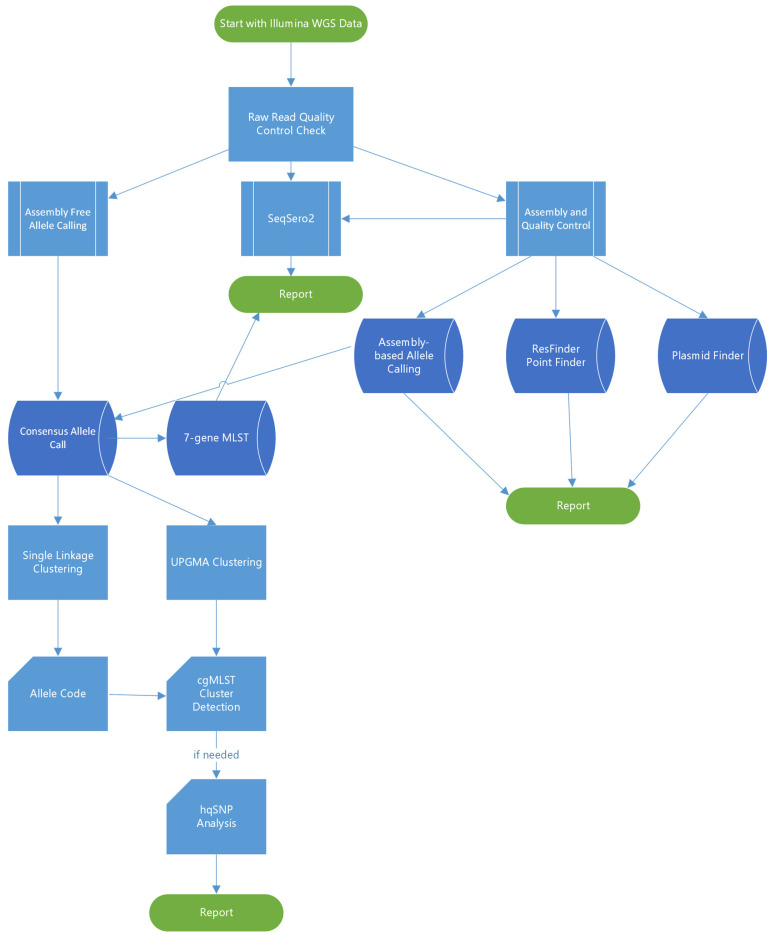
Summary of the bioinformatics workflow used in the study.

The overall bioinformatics workflow is illustrated in Figure 1. All sequences were analyzed using the BioNumerics 7.6.3 software (Applied Maths, Sint-Martens-Latem, Belgium) which had been customized for the PulseNet workflow. Assemblies were generated with Spades v3.7.1 implemented in BioNumerics and analyzed against a local implementation of the Enterobase core genome multi-locus sequence typing (cgMLST) scheme for *Salmonella* [15,16]. Alleles were detected using assembly-free and assembly-based methods. Assembly-free allele calls required a minimum of 3× coverage with reads in both forward and reverse orientation and 100% match to existing approved alleles in the cgMLST database. Assembly-based allele calls required a minimum of 75% similarity to existing approved alleles and the presence of start and stop codons. Only sequences passing the PulseNet critical quality metrics (minimum average coverage ≥ 30×, minimum average Phred score ≥ 30, percentage of core genome detected ≥85%, assembly size 4.40–5.69 MB, secondary species abundance ≤ 1.0) and having a matching serotype prediction (a local BioNumerics implementation of SeqSero2 [17] to the conventional serotyping result were included in the study. Seven-gene MLST sequence types (ST) were determined using PubMLST (https://pubmlst.org/, accessed on 1 January 2020). Phylogenetic trees were constructed based on categorical differences and unweighted pair group method with arithmetic mean (UPGMA) clustering. PulseNet cgMLST allele codes were automatically assigned using a hierarchical single-linkage reference-based algorithm utilizing a normalized Hamming distance. For *Salmonella*, a six-digit allele code was assigned with each digit corresponding to discrete allelic differences separating clusters (80, 28, 15, 7, 4 and 1 allele differences). The fourth digit, corresponding to approximately seven allele differences, is considered most useful for *Salmonella* cluster detection. The allele codes for this study were assigned in October 2021 and are subject to change, due to allele code merging events that occur in clonal serovars when two or more allele codes become impossible to separate from each other as genomes that fall in between the two codes are added. 

The NARMS workflow for detection of AR and plasmid profiles was performed outside BioNumerics. De novo assemblies were generated using shovill v1.0.9 (https://github.com/tseemann/shovill) (accessed on 1 February 2023), and contigs with coverage of below 10% average genome coverage were excluded. AR determinants were detected using staramr, v.0.4.0 [18], which employed the ResFinder database (updated 30 July 2020; 90% identity, 50% gene coverage) and the *Salmonella* subsp. PointFinder scheme (updated 30 August 2019). Plasmid targets were detected using a database adapted from PlasmidFinder [19] (90% identity, 60% gene coverage). Predicted AR was based on the phenotype.txt files provided by the Center for Genomic Epidemiology for both ResFinder and PointFinder (https://cge.food.dtu.dk/services/ResFinder/) (accessed on 1 February 2023). When necessary, if resistance to multiple drugs within the same class was predicted, we chose the representative drug phenotypically tested by NARMS (Table S2). 

High-quality single nucleotide polymorphism (hqSNP) analysis was performed for 18 sequences from seven outbreaks, showing increased variation in the cgMLST analysis using the lyve-SET version 1.1.4f [20] implemented in Terra.Bio (Theiagen, Highlands Ranch, CO, USA) with the following settings: allowedFlanking 5, min_alt_frac 0.95, min_coverage 20, phages and cliffs masked. Spades assemblies exported from BioNumerics were used as internal reference sequences for each of the seven outbreaks. Twenty-five hqSNPs were used as a cut-off to define non-matching isolates (i.e., polyclonal outbreak). This cut-off is more conservative than the 20 SNPs suggested by Wang et al. [21] for investigating *Salmonella* in food production facilities. However, it is in accordance with the SNP thresholds utilized in the ‘SNP addresses’ of the SnapperDB developed by Dallman et al. [22] for foodborne disease surveillance in England. 

## 3. Results

### 3.1. General Description of the Outbreaks

The one hundred outbreaks included in the study were caused by 35 different *Salmonella* serovars, 15 of which belong to the top 20 most common serovars in the USA. Outbreaks caused by the serovars Newport (17), Typhimurium (13), Enteritidis (7), Montevideo (6), I 4,[5],12:i:- (5), Heidelberg (5), and Javiana (5) were most frequent, while over half of the serovars were represented by a single outbreak (Table 1). Up to 71 of the outbreaks were classified as resulting from foodborne transmission and 29 were linked to contact with live animals (i.e., zoonotic). Among foodborne outbreaks, the most frequent vehicle categories were vegetables (20), poultry (10), meat (9), and fruit (8) (Table 1).

### 3.2. Diversity of cgMLST Profiles

**Figure 2:**
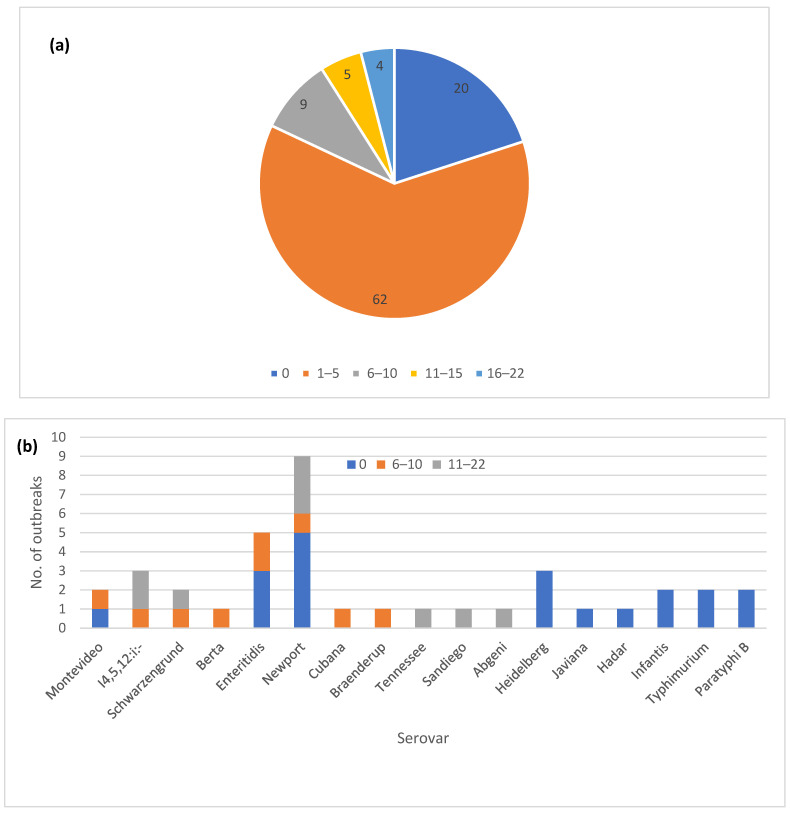

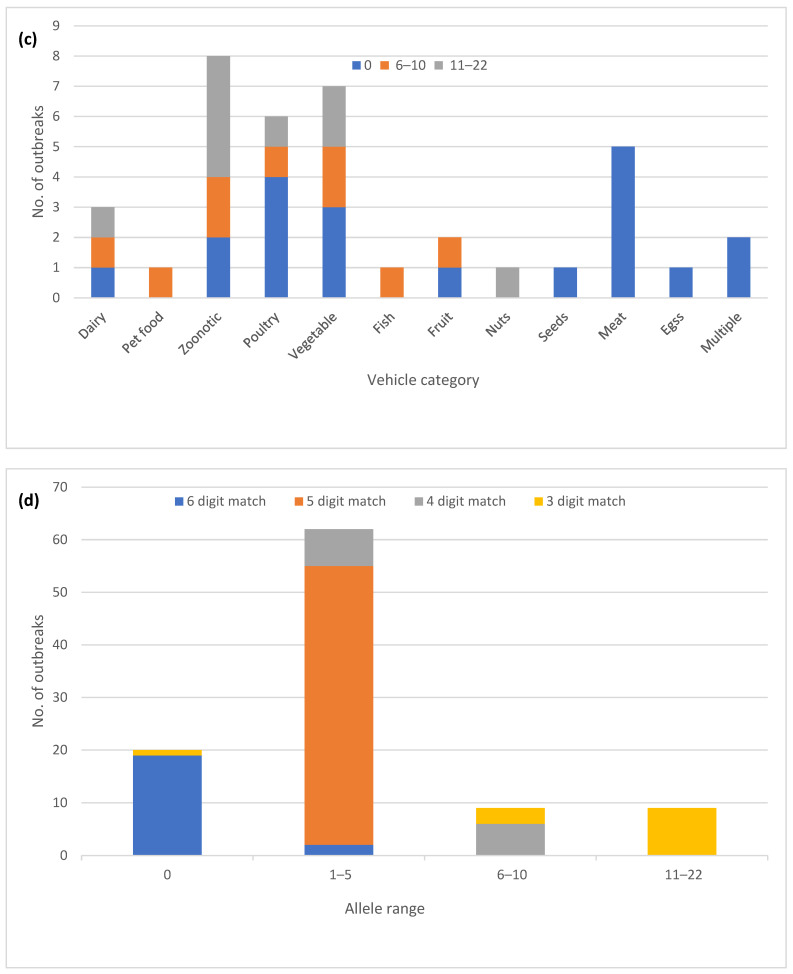
Core genome multi-locus sequence type (cgMLST) allele variation in one hundred *Salmonella enterica* subsp. *enterica* outbreaks: (**a**) Proportion of outbreaks in different allele range categories; (**b**) Outbreaks with zero allele difference and >5 alleles differences by the serovar; (**c**) Outbreaks with zero allele difference and >5 allele differences by the vehicle type; (**d**) Correlation of the outbreak allele range with the matching digits in the allele code.

The one hundred outbreaks were placed in five different allele range categories based on the highest observed allele difference between the sequenced isolates for each outbreak (Figure 2a). The majority of the outbreaks (82) showed five or fewer allele differences between the isolates. An additional nine outbreaks fell into the 6–10 allele difference category and in the remaining nine outbreaks, the isolates differed by 11–22 alleles. Analyzing a higher number of sequences from an outbreak did not appear to increase the level of variation. The nine outbreaks with most allele variation included six different serovars, with Newport (three) being most frequent (Figure 2b), while four were classified as zoonotic outbreaks and five were classified as foodborne (Figure 2c). 

The automated allele code assignment system used by PulseNet USA for initial triaging of clusters matched well with the observed allele differences (Figure 2d). Up to 87 out of the 91 outbreaks with 10 or fewer allele differences also had at least four matching digits in the allele code. The exceptions were three outbreaks in the 6–10 allele category where at least one isolate matched to the third allele code digit, and one outbreak with zero allele differences that was assigned only a three-digit allele code due to allele code merging. 

In six instances, the same strain caused two different outbreaks that were temporally separated by 5 to 23 months (Table S1). These outbreaks involved the serovars Braenderup, I 4,[5],12:i:-, Johannesburg, Montevideo, Schwarzengrund, and Typhimurium. In five instances, repeat outbreaks were associated with live poultry contact and the sixth was caused by exposure to live or frozen rodents used as petfood. The serovar Schwarzengrund and I 4,[5],12:i:- strains showed higher variation at 2–11 and 4–14 allele differences, respectively, while the other four outbreak strains varied by less than five allele differences.

### 3.3. Confirmatory hqSNP Analysis

Seven outbreaks containing a total of 18 isolates were not considered clusters based on the observed cgMLST allele range and the assigned allele code (Table 2). We used hqSNP analysis to confirm the cgMLST observations (Table S3). In four out of seven outbreaks, the hqSNP variation was in the range of 7–24 SNPs, corresponding to slightly increased variation (up to 15 allele differences) seen with cgMLST (Table 2). In the remaining three outbreaks with up to 22 alleles variation, the highest SNP counts were in the range of 38–47 SNPs, indicating the presence of multiple strains within the outbreaks. These three polyclonal outbreaks were caused by serovars Newport (2) and I 4,[5],12:i:- and were associated with the consumption of tomatoes (2) and breaded stuffed chicken (Table 2).

### 3.4. Correlation of cgMLST and PFGE

Most of the outbreaks (95/100) included only a single PFGE pattern (Table S1). The five outbreaks with multiple closely related PFGE patterns involved zoonotic outbreaks of the serovars Altona, Kiambu, and Montevideo, a poultry-related outbreak of serovar Heidelberg, and a beef-associated outbreak of serovar Newport. In all five outbreaks, the cgMLST variation was 0–5 allele differences.

### 3.5. Diversity of 7-Gene MLST

In all one hundred outbreaks, only a single 7-gene MLST type was detected in each outbreak. Among the 438 strains included in the study, 5 strains from five different outbreaks did not have a 7-gene MLST assigned (Table S1). These five outbreaks were caused by five different serovars (Agbeni, Braenderup, Montevideo, Newport, Typhimurium) and two of them were zoonotic. On three occasions, no allele was assigned in one of the seven loci because there was a discrepancy between assembly-based and assembly-free allele assignment methods, in each case caused by a single nucleotide mutation. In one case, all seven loci were assigned alleles, but the profile was new to the public database, therefore no sequence type was assigned. The new profile differed from the outbreak profile by a single nucleotide mutation at one locus. In the fifth case, no sequence type was assigned because there were discrepancies between the assembly-based and assembly-free allele assignment methods at three loci caused by multiple mutations.

### 3.6. Diversity of AR

**Figure 3:**
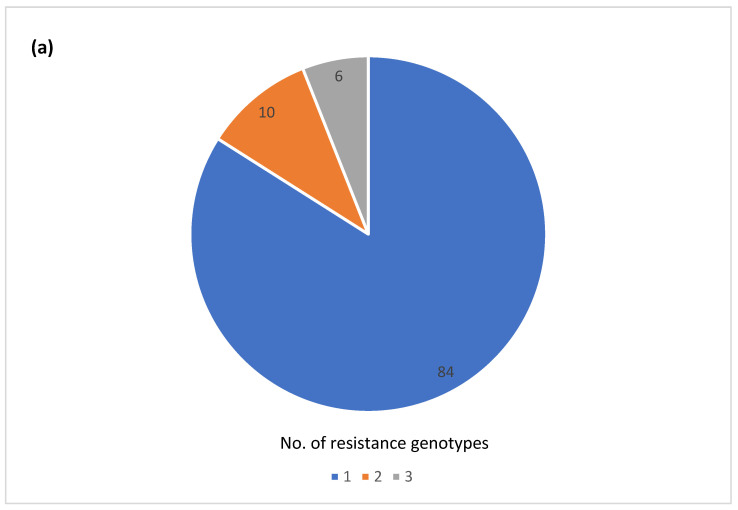

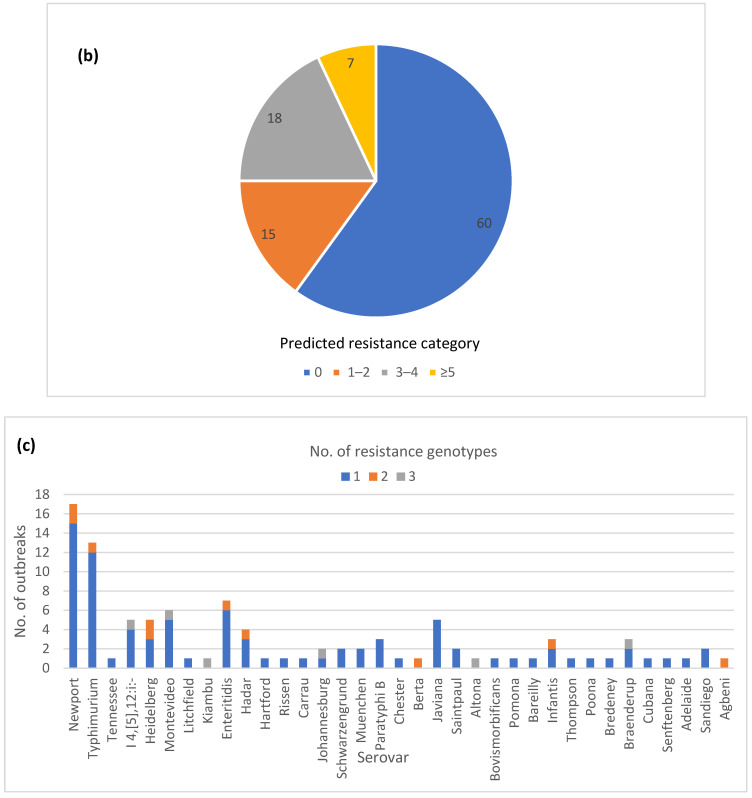

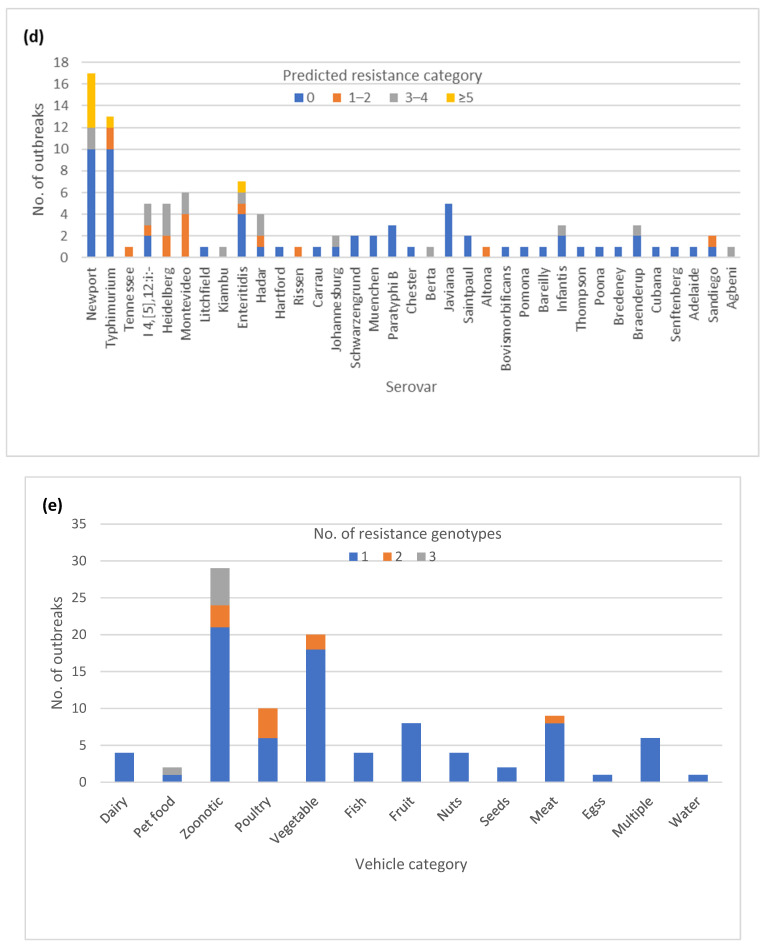

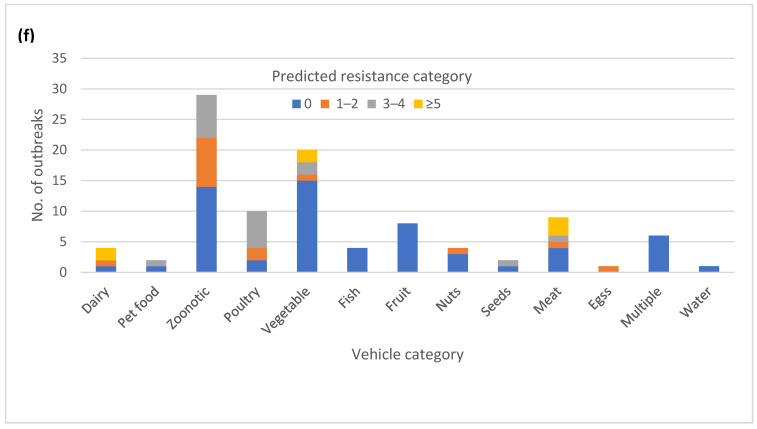
Antimicrobial resistance (AR) genotype profile variation in one hundred *Salmonella enterica* subsp. *enterica* outbreaks: (**a**) Proportion of outbreaks with clonal or variable AR genotype profiles. Pan-susceptible outbreaks were categorized as having a single AR genotype; (**b**) Proportion of outbreaks belonging to different resistance categories. Resistance categories were defined based on the number of antibiotic classes with predicted resistance. Outbreaks with multiple resistance profiles were categorized based on the most resistant isolate; (**c**) Number of AR genotypes per outbreak by the serovar (**d**) Number of outbreaks in different predicted resistance categories by the serovar; (**e**) Number of AR genotypes per outbreak by the vehicle type; (**f**) Number of outbreaks in different predicted resistance categories by the vehicle type.

The majority of the outbreaks (84) had no variation in AR genotype, with outbreaks being either pan-susceptible (60) or harboring a single AR genotype (24) (Figure 3a). The remaining outbreaks (16) had two or three different AR genotypes. Twenty-five outbreaks could be classified as multi-drug resistant (MDR) by at least one isolate, having predicted resistance to the three or more antibiotic classes (Figure 3b). A total of 7 out of the 25 outbreaks had predicted resistance to five or more antibiotic classes, but none fit our current definition for extensive drug resistance, which was defined here as resistance to ampicillin, amoxicillin, azithromycin, ciprofloxacin and cotrimoxazole. Fourteen of the 25 MDR outbreaks had multiple different AR genotypes among the isolates (Table S1). Variation in AR genotypes was not unique to a specific serotype (Figure 3c). The majority of the MDR outbreaks occurred in serovars Newport, Heidelberg, I 4,[5],12:i-, Montevideo, Enteritidis, and Hadar (Figure 3d). The variable AR genotypes as well as the predicted MDR profiles occurred mainly in zoonotic and foodborne poultry-associated outbreaks (Figure 3e,f).

### 3.7. Diversity of Plasmid Profiles

**Figure 4:**
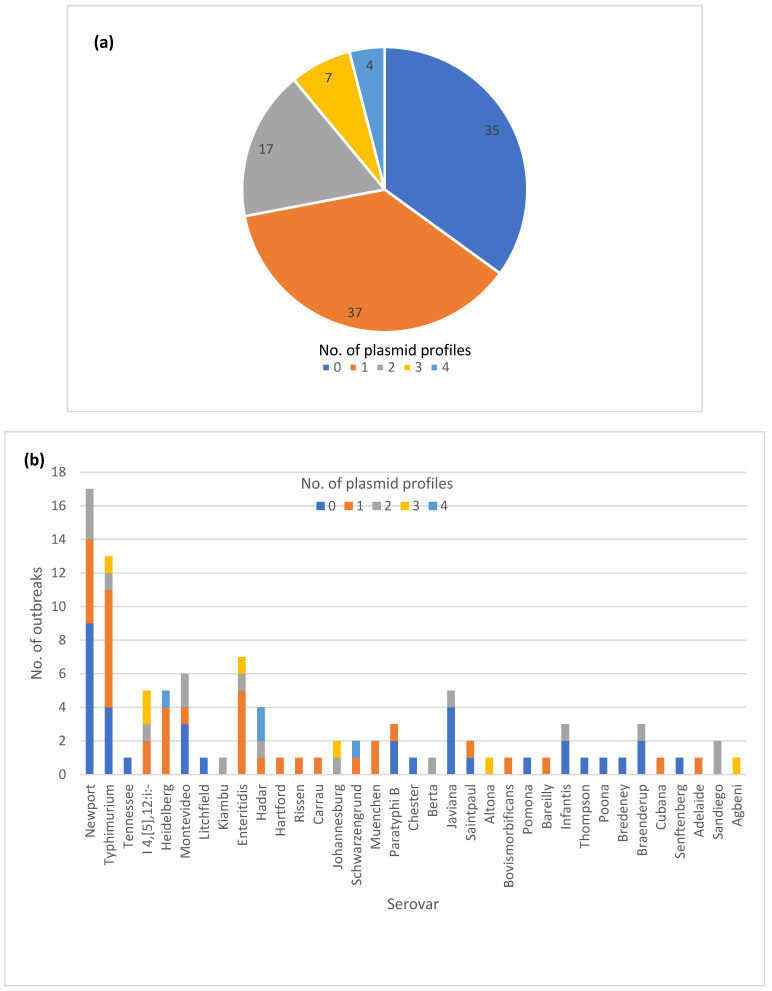

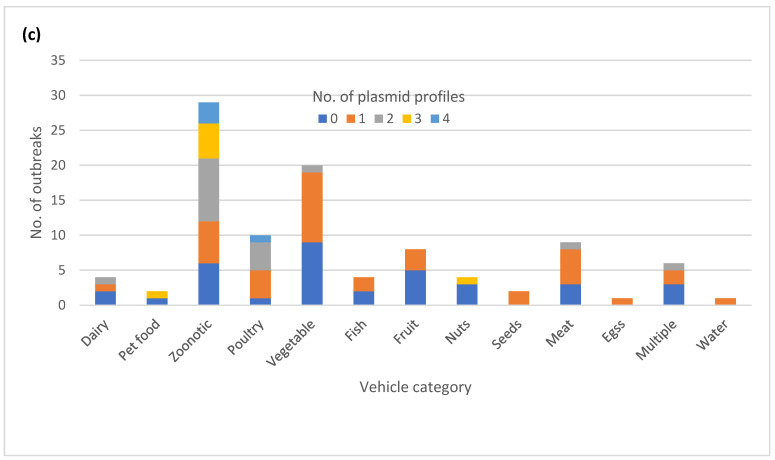
Plasmid profile variation in one hundred *Salmonella enterica* subsp. *enterica* outbreaks: (**a**) Proportion of outbreaks with clonal or variable plasmid profiles. No plasmids detected were categorized as its own plasmid profile; (**b**) Number of outbreaks in different plasmid profile categories by the serovar; (**c**) Number of plasmid profiles per outbreak by the vehicle category.

The majority of the outbreaks had no variation in plasmid profiles, with either no plasmids detected (35) or a single plasmid profile (37) (Figure 4a). The isolates in the remaining outbreaks (28) had two to four plasmid profiles identified. A total of 14 out of 16 outbreaks with variable AR genotypes also exhibited variation in plasmid profiles. Thirteen out of 25 MDR outbreaks had variation in plasmid profiles. (Table S1). The variable plasmid profiles were distributed among multiple serotypes (Figure 4b), with the most common vehicle categories being zoonotic and foodborne poultry associated (Figure 4c).

### 3.8. Correlation of cgMLST Clustering with AR and Plasmid Profiles for Selected Serovars

**Figure 5:**
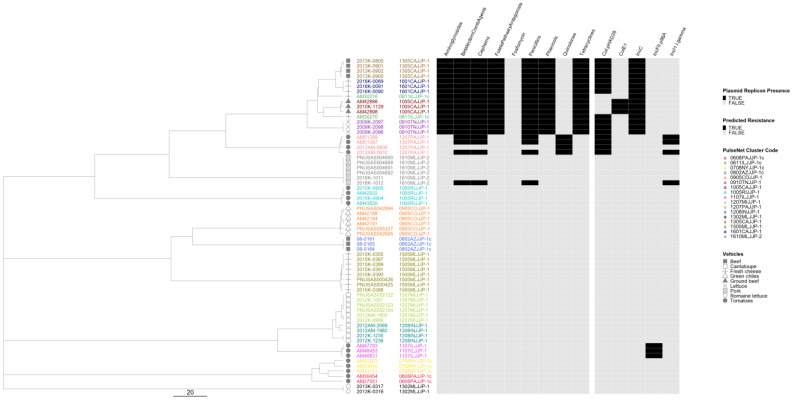
*Salmonella enterica* subsp. *enterica* serovar Newport phylogeny based on core genome multi-locus sequence (cgMLST) typing for the 17 outbreaks included in the study with antimicrobial resistance (AR), plasmid and vehicle category data overlayed. A black square corresponds to presence of AR determinants and/or plasmids; a white square corresponds to absence of AR determinants and/or plasmids. Different outbreaks are color-coded. Different vehicles are indicated by unique icons. The phylogenetic tree was constructed based on categorical differences and unweighted pair group method with arithmetic mean (UPGMA) clustering. The scale corresponds to the number of allele differences.

We generated separate cgMLST dendrograms for five serovars and overlayed AR and plasmid data and vehicle information to assess possible associations between genotypes and outbreak vehicles. For serovar Newport, 16 out of 17 outbreaks formed tight clusters clearly separating from each other (Figure 5). Two isolates from the remaining outbreak differed from each other by 15 alleles and clustered as singletons. All five Newport outbreaks with predicted resistance to five or more antibiotic classes clustered in the same sub-lineage with no other outbreaks in it, and all contained an IncC plasmid replicon. The two other MDR outbreaks clustered adjacent to this highly resistant lineage. Overall, the presence of plasmids correlated with the presence of AR. Only one outbreak, linked to consumption of tomatoes, exhibited a variation in AR genotypes which appeared to be linked to the presence or absence of the IncI1-I(gamma) plasmid replicon. 

**Figure 6:**
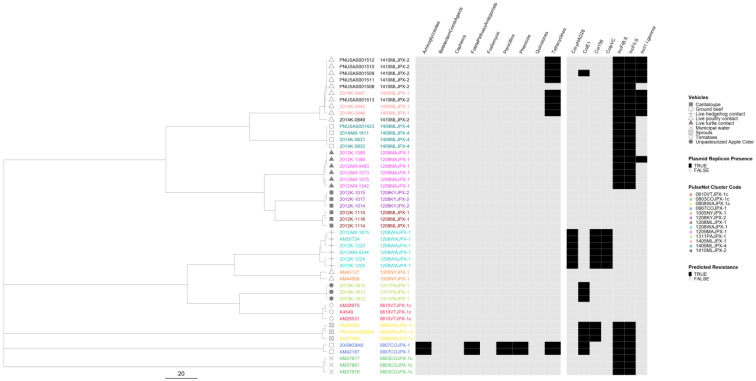
*Salmonella enterica* subsp. *enterica* serovar Typhimurium phylogeny based on core genome multi-locus sequence (cgMLST) typing for the 13 outbreaks included in the study with antimicrobial resistance (AR), plasmid and vehicle category data overlayed. A black square corresponds to presence of AR determinants and/or plasmids; a white square corresponds to absence of AR determinants and/or plasmids. Different outbreaks are color-coded. Different vehicles are indicated by unique icons. The phylogenetic tree was constructed based on categorical differences and unweighted pair group method with arithmetic mean (UPGMA) clustering. The scale corresponds to the number of allele differences.

In the serovar Typhimurium phylogeny, 11 out of 13 outbreaks were well separated from each other (Figure 6). Isolates from two live poultry-associated outbreaks were intertwined, and the epidemiological investigation indicated a common source for the implicated poultry. Only three outbreaks were predicted to be resistant, of which one was classified as MDR. The two outbreaks with predicted tetracycline resistance clustered in the same lineage and the MDR outbreak formed a lineage of its own. Plasmids were detected in nine outbreaks, including six pan-susceptible outbreaks. Only two zoonotic outbreaks showed minor variation in plasmid profiles and no variation was detected in the AR profiles.

**Figure 7:**
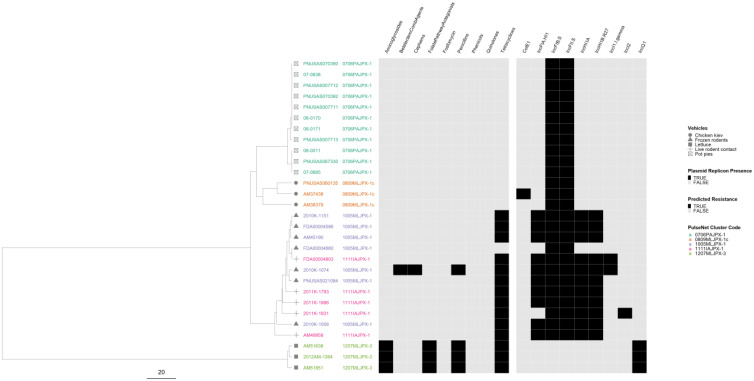
*Salmonella enterica* subsp. *enterica* serovar I 4,[5],12:i:- phylogeny based on core genome multi-locus sequence (cgMLST) typing for the five outbreaks included in the study with antimicrobial resistance (AR), plasmid and vehicle category data overlayed. A black square corresponds to presence of AR determinants and/or plasmids; a white square corresponds to absence of AR determinants and/or plasmids. Different outbreaks are color-coded. Different vehicles are indicated by unique icons. The phylogenetic tree was constructed based on categorical differences and unweighted pair group method with arithmetic mean (UPGMA) clustering. The scale corresponds to the number of allele differences.

In the serovar I 4,[5],12:i:- phylogeny, isolates from three outbreaks were separated from each other, while the isolates from the remaining two outbreaks were intertwined (Figure 7). The two intertwining outbreaks were caused by contact with live or frozen rodents that originated from the same source. The three AR outbreaks clustered together in two sub-lineages, separate from the pan-susceptible outbreaks. All isolates had plasmids, but genomes from the two rodent-associated outbreaks had up to six plasmids and displayed variation in their plasmid content. One genome in one of the two rodent-associated outbreaks displayed predicted resistance to additional drug classes. One of the MDR outbreaks had an IncQ1 plasmid target not seen in any of the other outbreaks.

**Figure 8:**
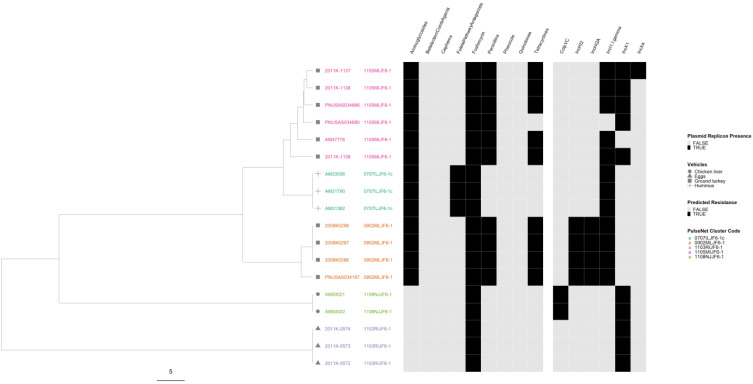
*Salmonella enterica* subsp. *enterica* serovar Heidelberg phylogeny based on core genome multi-locus sequence (cgMLST) typing for the five outbreaks included in the study with antimicrobial resistance (AR), plasmid and vehicle category data overlayed. A black square corresponds to presence of AR determinants and/or plasmids; a white square corresponds to absence of AR determinants and/or plasmids. Different outbreaks are color-coded. Different vehicles are indicated by unique icons. The phylogenetic tree was constructed based on categorical differences and unweighted pair group method with arithmetic mean (UPGMA) clustering. The scale corresponds to the number of allele differences.

In the serovar Heidelberg phylogeny, all five outbreaks were well separated from each other with the three MDR outbreaks forming their own lineage (Figure 8). All isolates were predicted to be resistant to at least one drug class, and all contained at least one plasmid replicon. Only one outbreak, associated with the consumption of ground turkey, showed variation in both AR and plasmid profiles.

**Figure 9:**
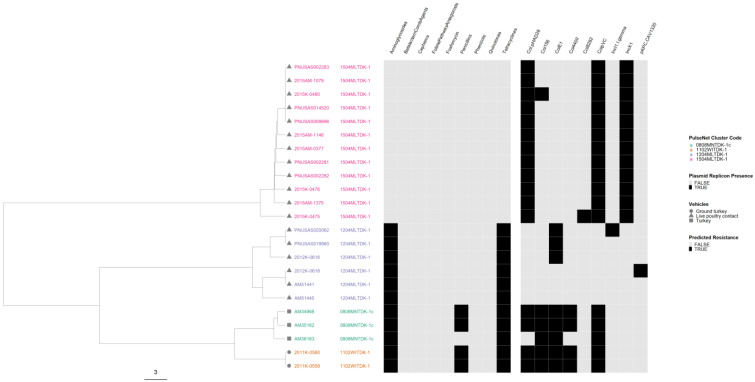
*Salmonella enterica* subsp. *enterica* serovar Hadar phylogeny based on core genome multi-locus sequence (cgMLST) typing for the four outbreaks included in the study with antimicrobial resistance (AR), plasmid and vehicle category data overlayed. A black square corresponds to presence of AR determinants and/or plasmids; a white square corresponds to absence of AR determinants and/or plasmids. Different outbreaks are color-coded. Different vehicles are indicated by unique icons. The phylogenetic tree was constructed based on categorical differences and unweighted pair group method with arithmetic mean (UPGMA) clustering. The scale corresponds to the number of allele differences.

In the serovar Hadar phylogeny, all four outbreaks were well separated from each other (Figure 9). The three AR outbreaks clustered in the same lineage, with the two MDR outbreaks forming their own sub-lineage. Variation in plasmid profiles occurred in three outbreaks, but only one of them, again associated with consumption of ground turkey, also displayed variation in AR profiles.

## 4. Discussion

### 4.1. Diversity of cgMLST Profiles and Confirmatory hqSNP Analysis

According to the PulseNet cluster definition guidance for the local level (e.g., state), a *Salmonella* cluster should be assigned and monitored if there are three or more isolates within 10 allele differences and at least two of those isolates are within five allele differences within a 60-day window [5]. When cgMLST allele variation was evaluated in our dataset of 100 outbreaks, 18 had variation above five alleles but 11 of these also included closely related isolates to still fit the cluster definition based on the allele range. At the national level, PulseNet used an allele code system to aid the initial triaging of clusters. Groups of isolates with four matching digits in the six-digit allele code are further evaluated by visualizing them in a phylogenetic tree. A total of 87 out of 100 outbreaks had four matching digits in the allele code. Six additional outbreaks with only three-digit matches would have been considered a cluster based on the allele range, leaving seven outbreaks that would have been missed if only cgMLST data with strict cluster definition rules was used for cluster detection. A confirmatory hqSNP analysis showed that four out of the seven outbreaks appeared to have been caused by a single strain with increased variation (less than 25 SNPs). The remaining three outbreaks were deemed polyclonal, caused by two genetically distinct strains differing by 38–47 SNPs. This illustrates the fact that while cgMLST data can be used as a starting point for triaging clusters of clinical illness for epidemiological investigations, additional data measuring genetic relatedness may sometimes be helpful in deciding whether a cluster merits an epidemiological investigation. The strictest possible cluster detection criteria based on genomic data should be used to define illness clusters in order not to confound the epidemiological signal [8]. Outbreak case definitions, on the other hand, can have allele difference ranges exceeding the cluster detection thresholds if the epidemiologic data support using wider genomic case criteria. However, our data also suggests that, in some instances, epidemiological investigations may be warranted even though the genomic data do not indicate high genetic relatedness between isolates. 

In our dataset, serovars I 4,[5],12:i:- and Newport were overrepresented in clusters showing increased cgMLST variation. Serovar Newport was also overrepresented in clusters showing zero allele variation, together with serovars Heidelberg and Enteritidis. Given the polyphyletic nature of serovar Newport, it is possible that one lineage evolves faster than the other and it may not be surprising to find serovar Newport in both the clonal and the highly variable groups. The study conducted by Pightling et al. [23] proposed 1.97 SNPs per year as an average evolutionary rate for *Salmonella* when considering genome sequence match criteria. In their study, serovars Heidelberg and Enteritidis evolved below the average rate in accordance with our results. However, the two lineages of Newport included in the Pightling et al. study did not exhibit evolutionary clock-like behavior, and serovar I 4,[5],12:i:- was not included [23]. 

Zoonotic outbreaks were overrepresented in our dataset as a vehicle for clusters showing increased cgMLST variation, whereas meat and poultry consumption were overrepresented as vehicles for clusters with no variation. However, of the seven outbreaks that would not have been recognized using cgMLST data alone, only two were zoonotic, and none of the three outbreaks considered polyclonal were zoonotic. The higher variation in zoonotic outbreaks may be explained by in vivo evolution of the bacteria in the host animal that is accentuated by animal-to-animal and/or animal-to-person transmission. Zoonotic outbreaks also tend to last for a relatively long time compared to outbreaks associated with food commodities, due to persistence of the outbreak strain in the host animal population, which gives the outbreak strain time to evolve [24,25]. 

Our dataset also included six instances where the same strain caused two temporally separated outbreaks, suggesting persistence of the source of the outbreaks in question. Five instances of these reoccurring outbreaks were associated with exposure to live poultry and one with exposure to the rodents used to feed pet reptiles. Reoccurring *Salmonella* outbreaks associated with exposure to live poultry, particularly baby poultry originating in mail-order hatcheries and agricultural feed stores, are a well-documented public health issue in the USA [26,27,28,29]. *Salmonella* strains entering the hatcheries with incoming breeder birds can become persistent residents within the hatchery, especially without a stringent *Salmonella* control program and biosecurity measures [26]. Similarly, establishments raising feeder rodents can become contaminated by persistent resident *Salmonella* strains, as rodents are natural hosts for *Salmonella*. The repeat outbreaks caused by serovar I 4,[5],12:i:- in feeder rodents included in this study and originating from a single provider were described by Cartwright et al. [30]. PulseNet USA has also started formally tracking reoccurring, emerging and persisting strains to respond more quickly to these types of outbreaks (https://www.cdc.gov/ncezid/dfwed/outbreak-response/rep-strains.html) (accessed on 1 April 2024).

### 4.2. Correlation of cgMLST with Legacy Subtyping Methods 

We also evaluated variations within our dataset using the two legacy subtyping methods, PFGE and 7-gene MLST. By PFGE, only 5 outbreaks out of 100 exhibited multiple PFGE patterns. However, the PFGE variation did not correlate with the cgMLST variation, with all five outbreaks belonging to the 0–5 allele category. The slight variation in PFGE patterns seen in outbreaks is typically caused by recombination of the genome and mobile elements such as plasmids [31], not by single nucleotide mutations that affect cgMLST allele differences. A recombination event typically causes only a single allele difference in cgMLST [32], unless it involves a very large section and multiple genes of the genome. Additionally, mobile elements are excluded from the cgMLST scheme because they can confound phylogenetic signals through inflated genetic variation [16]. It can be argued, therefore, that cgMLST data are epidemiologically more concordant than the PFGE data. The seven-gene MLST data, on the other hand, are known to have discriminatory power equal to serotyping which was confirmed in our study. The only variations detected were caused by technical issues stemming from the allele assignment process in BioNumerics, many of which were likely caused by sequencing errors in the data.

### 4.3. Diversity of AR and Plasmid Profiles

The variation in AR genotypes occurred mainly in MDR outbreaks that exhibited predicted resistance for at least three antibiotic classes. As expected, variation in AR genotypes coincided with variation in plasmid profiles. Only 2 of the 16 outbreaks with AR profile variation did not show variation in plasmid profiles. Variation in plasmid profiles in AR variable outbreaks was mainly caused by presence/absence of IncI1, IncI2, IncF, IncH, and IncX replicons that are known to be associated with MDR [33]. On the other hand, half of the 28 outbreaks with variable plasmid profiles of various replicon types did not show any variation in AR profiles. Plasmids that do not confer AR also play an important part in the evolutionary capacity in *Salmonella* populations and therefore variation driven by factors other than antibiotic selection pressure can be expected to occur [34]. Not surprisingly, variation in AR profiles did not coincide with variation in cgMLST as only 1 of the 16 AR variable outbreaks showed variation above 10 alleles. Of the 28 outbreaks demonstrating plasmid variation, 5 showed variation above 10 alleles in cgMLST, and 3 of those 5 would not have been considered a cluster based on the PulseNet cluster detection rules. However, only one of the three was deemed polyclonal based on hqSNP analysis. The poor correlation of cgMLST and plasmid and AR profiles is an expected finding as mobile elements are not included as targets in the cgMLST scheme [16].

While there was no clear association with the variable AR and plasmid profiles to certain serotypes, zoonotic, and foodborne poultry-associated outbreaks were clearly overrepresented for both AR and plasmid profile variation, as well as for MDR outbreaks. Other studies have also reported plasmid-mediated AR in outbreaks caused by meat and poultry products, as well as in zoonotic outbreaks caused by contact with live poultry and cattle [35,36,37,38,39]. More work is needed to understand the impact of antibiotic use and other management practices on AR and plasmid variability in food production animal reservoirs [40]. In addition to food production animals, salmonellosis outbreaks with AR have also been reported after contact with household pets [41] and their animal-derived food [30,42].

### 4.4. Correlation of cgMLST Clustering with AR and Plasmid Profiles for Selected Serovars

When AR and plasmid data was overlayed on the phylogenies based on the cgMLST data for five common serovars, the MDR strains clustered in their own lineages away from the pan-susceptible strains. Similarly, when Turcotte et al. [43] examined the *S. enterica* population structure in New Hampshire, USA, they found that the MDR strains of each serovar formed their own lineages. Lee et al. [44] also reported that the majority of the *Salmonella* ser. Infantis strains in England and Wales carrying the pESI-like plasmid conferring MDR belonged to a single phylogenetic clade. These observations are all likely examples of clonal expansion where a less dominant strain acquires mobile elements carrying resistance and other determinants, giving it a selection advantage to outcompete other strains in the same lineage. While looking further into associations between AR and plasmid profiles, it was notable that in serovar Newport, the presence of plasmids was almost exclusively associated with AR with the exception of one outbreak. In the serovars Typhimurium, I 4,[5],12:i:-, Heidelberg and Hadar, almost all strains possessed plasmids but only some plasmids appeared to be associated with resistance.

### 4.5. Study Limitations

This study has some limitations. Due to the availability of strains for sequencing from cases with confirmed epidemiological data, particularly from older outbreaks, many outbreaks were represented by a limited number of isolates. Including additional isolates could have changed the categorization of some outbreaks from clonal to variable. Conversely, if additional isolates were sequenced from the seven outbreaks that did not fit the PulseNet cluster definition rules, more closely related isolates could have been detected that may have changed the conclusions for those outbreaks. Additionally, only 35 *Salmonella* serovars were included in the study and some common outbreak vehicle categories, such as eggs, were poorly represented. Therefore, some associations between serotypes, vehicles, and genetic variation may have been missed. Some variation observed in the study, particularly those associated with the presence/absence of plasmids and AR determinants, may not have been true genetic variations but instead caused by technical limitations stemming from the inability of the short read sequencing data to adequately capture plasmids [44,45].

## 5. Conclusions

In our study, one hundred *Salmonella* outbreaks representing 35 serovars and 13 broad vehicle categories with 36 different vehicle/exposure types were evaluated for genetic variation. Based on our results, a close allele range (less than ten allele differences with a subset closer than five allele differences) in cgMLST profiles can be considered a reliable proxy for epidemiological relatedness for the majority of outbreak investigations. However, it is important to recognize that the serovar and the vehicle type may impact the variability in cgMLST allele differences. Therefore, it is critical to consider both the WGS and the epidemiological data to develop a case definition that accurately groups together ill people that share a common source. Variation in the AR and plasmid profiles was more common than variation in cgMLST profiles and showed affinity to certain vehicle types. Mobile elements contain valuable information about the selective pressures that bacterial populations are undergoing [46]; however, they are often difficult to interpret and can confound core genome phylogenetic signals. More research is needed regarding how to incorporate mobile elements into our understanding of the epidemiology of foodborne and zoonotic outbreaks caused by *Salmonella enterica*.

## Figures and Tables

**Table 1:** Summary of the *Salmonella enterica* spp. *enterica* isolates and outbreaks included in the study. ND = not determined.

Serovar	No. of Isolates	No. of Outbreaks	cgMLST Allele Range	7-Gene MLST	Vehicle Category (Vehicle)
**Adelaide**	2	1	3	440	Meat (pork)
**Agbeni**	24	1	0–21	2606, ND	Zoonotic (turtles)
**Altona**	4	1	1–4	1549	Zoonotic (live poultry)
**Bareilly**	4	1	0–2	909	Fish (raw tuna scrape)
**Berta**	3	1	5–7	435	Poultry (ground turkey)
**Bovismorbificans**	2	1	2	377	Seeds (hummus)
**Braenderup**	16	3	1–2	22, ND	Zoonotic (live poultry)
			0–2	22	Zoonotic (live poultry)
			0–6	22	Zoonotic (live poultry)
**Bredeney**	4	1	1–4	505	Nuts (peanut butter)
**Carrau**	4	1	0–2	226	Fruit (melon)
**Chester**	5	1	0–2	2063	Multiple (frozen dinners)
**Cubana**	4	1	2–6	286	Vegetable (sprouts)
**Enteritidis**	28	7	0	11	Zoonotic (reptile/live rodent)
			2–3	11	Vegetable (sprouts)
			0–2	11	Nuts (Turkish pine nuts)
			1–6	11	Vegetable (lettuce)
			3–6	11	Fish (tuna sushi)
			0	11	Poultry (chicken)
			0	11	Poultry (breaded stuffed chicken)
**Hadar**	23	4	1–2	33	Poultry (turkey)
			0	33	Poultry (ground turkey)
			0–5	33	Zoonotic (live poultry)
			0–4	33	Zoonotic (live poultry)
**Hartford**	2	1	2	405	Vegetable (salsa)
**Heidelberg**	18	5	0	15	Seeds (hummus)
			0–2	15	Poultry (ground turkey)
			0	15	Eggs (custard-filled pastry)
			1–5	15	Poultry (ground turkey)
			0	15	Poultry (chicken liver)
**I 4,[5],12:i:-**	29	5	0–4	19	Multiple (pot pies)
			10–22	19	Poultry (breaded stuffed chicken)
			0–10	19	Pet food (frozen rodents)
			4–14	19	Zoonotic (rodents)
			2–4	34	Vegetable (lettuce)
**Infantis**	10	3	3–5	32	Pet food (dog food)
			0	32	Zoonotic (live poultry)
			0	32	Meat (pork)
**Javiana**	58	5	0–1	24	Vegetable (tomatoes)
			0	24	Vegetable (tomatoes)
			0–1	24	Vegetable (cucumbers)
			0–3	24	Fish (tilapia)
			0–4	24	Multiple (potato salad)
**Johannesburg**	7	2	0–1	471	Zoonotic (live poultry)
			0–5	471	Zoonotic (live poultry)
**Kiambu**	3	1	0–2	309	Zoonotic (live poultry)
**Litchfield**	5	1	0–1	214	Fruit (cantaloupe melon)
**Montevideo**	15	6	7	138, ND	Dairy (shredded cheese)
			1	316	Poultry (unspecified)
			0	81	Meat (pork)
			4	4	Zoonotic (live poultry)
			2	4	Zoonotic (live poultry)
			0–5	316	Zoonotic (live poultry)
**Muenchen**	7	2	0–2	112	Fruit (blueberries)
			3–5	83	Zoonotic (live poultry)
**Newport**	66	17	20	118	Vegetable (tomatoes)
			15	45	Dairy (queso fresco-type cheeses)
			5–13	118	Vegetable (tomatoes)
			0	118	Meat (beef)
			0–2	2362, ND	Vegetable (green chilies)
			0	45	Vegetable (lettuce)
			1–2	45	Meat (ground beef)
			0–1	45	Vegetable (tomatoes)
			0–1	118	Vegetable (tomatoes)
			0–1	118	Fruit (cantaloupe)
			1–3	45	Vegetable (tomatoes)
			0–7	118	Fruit (cantaloupe melon)
			0	5	Vegetable (romaine lettuce)
			0–2	45	Meat (beef)
			0–2	118	Dairy (queso fresco-type cheeses)
			0	45	Dairy (queso fresco-type cheeses)
			0	45	Meat (pork)
**Paratyphi B var. L(+) tartrate+**	8	3	0–1	43	Fish (ahi)
			0	43	Multiple (raw tuna)
			0	307	Multiple (raw fish)
**Pomona**	3	1	2	451	Zoonotic (turtles)
**Poona**	2	1	2	3095	Zoonotic (turtles)
**Rissen**	4	1	0–5	469	Vegetable (white/black pepper)
**Saintpaul**	7	2	1–2	50	Multiple (steak, spice rub)
			2–5	50	Vegetable (cucumbers)
**Sandiego**	5	2	6–13	20	Zoonotic (turtles)
			4	126	Zoonotic (turtles)
**Schwarzengrund**	7	2	2–11	96	Zoonotic (live poultry market)
			2–8	96	Zoonotic (live poultry market)
**Senftenberg**	3	1	0–1	185	Nuts (pistachios)
**Tennessee**	5	1	0–18	319	Nuts (peanut butter)
**Thompson**	3	1	1–2	26	Zoonotic (live poultry)
**Typhimurium**	48	13	1–3	19	Vegetable (tomatoes)
			1–2	19	Water (municipal water)
			1–3	19	Vegetable (sprouts)
			2	19, ND	Meat (ground beef)
			4	19	Zoonotic (live poultry)
			0–1	19	Fruit (cantaloupe melon)
			1–3	19	Fruit (cantaloupe melon)
			0–5	19	Zoonotic (hedgehogs)
			0–2	19	Zoonotic (turtles)
			0	19	Fruit (unpasteurized apple cider)
			1–2	19	Zoonotic (live poultry)
			0	19	Meat (ground beef)
			0–5	19	Zoonotic (live poultry)
**Total (35)**	**438**	**100**			

**Table 2:** Summary of the seven *Salmonella enterica* spp. *enterica* outbreaks that did not fit the PulseNet USA cluster definition based on the core genome multi-locus sequence typing (cgMLST) allele differences.

PulseNet Cluster Code *	Serovar	Vehicle	No. of Isolates	cgMLST Allele Range	hqSNP Range
0608PAJJP-1 **	Newport	Tomatoes	2	20	47
0611ILJJP-1	Newport	Queso fresco-type cheeses	2	15	24
0708NYJJP-1 **	Newport	Tomatoes	3	5–13	11–38
0802COJIX-1	Montevideo	Shredded cheese	2	7	10
0809MLJPX-1 **	I 4,[5],12:i:-	Breaded stuffed chicken	3	10–22	24–46
0907MLJM6-1	Schwarzengrund	Live poultry contact	3	2–11	7–16
1508MLJLX-1	Sandiego	Live turtle contact	3	6–13	7–18

* Cluster code nomenclature: the first 4 digits denote the year and month of cluster detection, the next 2–4 letters denote the state or county of cluster detection or ML for multi-state, the last 3 letters/numbers denote the serovar, followed by–#. If multiple outbreaks meet the same criteria, then # is changed from 1 to 2, 2 to 3, etc. For example, 0809MLJPX-1 represents the 1st multi-state Typhimurium outbreak detected in September 2008. ** Outbreak deemed polyclonal, i.e., caused by two or more genetically distinct strains.

## Data Availability

The raw sequences have been made available on the Sequence Read Archive (SRA) of the National Center for Biotechnology Information (NCBI) in the BioProject PRJNA230403. The accession numbers for the individual sequences are listed in Table S1. Detailed results of the analyses performed in BioNumerics and by the in-house pipelines of the National Antimicrobial Resistance Monitoring System (NARMS) are included in Table S1. The high-quality single nucleotide polymorphism (hqSNP) analysis results are included in Table S3.

## Supplementary Materials

The following supporting information can be downloaded at: https://www.mdpi.com/article/10.3390/microorganisms12081563/s1, Table S1: Detailed results for the 438 *Salmonella enterica* subsp. *enterica* strains included in the study; Table S2: Antimicrobial agents included in the National Antimicrobial Resistance Monitoring System (NARMS) predicted resistance results; Table S3: Detailed results for the 18 *Salmonella enterica* subsp. *enterica* strains from seven outbreaks that did not fit the PulseNet USA cluster definition based on the core genome multi-locus sequence typing (cgMLST) allele differences.
